# Serum ferritin levels and disease severity in psoriasis: A cross-sectional study

**DOI:** 10.1097/MD.0000000000045781

**Published:** 2025-11-07

**Authors:** Puhua Zhang, Qiuhe Song, Zhongcheng Hu, Linghua Liu, Yadan Cao, Fangfang Liao, Chunxia Zhou

**Affiliations:** a Department of Cardiology, Affiliated Hospital of Jiujiang University, Jiujiang, Jiangxi, China; b Department of Dermatology, Affiliated Hospital of Jiujiang University, Jiujiang, Jiangxi, China.

**Keywords:** C-reactive protein, ferrtin, psoriasis, psoriasis area severity index

## Abstract

Psoriasis is a chronic, complex, inflammatory skin disease that significantly impacts the quality of life of patients. Research has found excessive accumulation of iron in the skin tissue of psoriasis patients. However, no clinical studies have reported the relationship between serum ferritin (SF) and psoriasis. This cross-sectional study aimed to explore the correlation between SF levels and psoriasis. The study was conducted from June 2020 to December 2023 at the affiliated hospital of Jiujiang University, involving 105 psoriasis patients and 52 controls. Mild and severe disease was defined based on the Psoriasis Area Severity Index. Fasting SF levels were analyzed in blood samples. Compared with the control group, SF levels were significantly elevated in psoriasis patients. In severe psoriasis patients, SF levels were even higher. SF was positively correlated with Psoriasis Area Severity Index, C-reactive protein, and disease duration, with statistically significant differences. In the receiver operating characteristic curve analysis, the optimal cutoff value (area under the curve, sensitivity, specificity) for SF was 216.1 (0.74, 63.98%, 75.00%). High SF levels are associated with the severity of psoriasis. Dysregulation of iron metabolism may play a role in the development of psoriasis. SF levels serve as markers for the severity and duration of psoriasis. By measuring ferritin levels early in the disease process, we can adopt preventive strategies to better manage and improve the survival and quality of life of psoriasis patients.

## 1. Introduction

Psoriasis is an immune-mediated chronic, recurrent, inflammatory, systemic disease triggered by the interaction between individuals and their environment, characterized by scaly erythematous plaques or patches that are noncontagious.^[1]^ The prevalence of psoriasis varies between 0.09% and 11.43% across different countries,^[2]^ making it a significant global issue. The core of psoriasis is immune dysregulation leading to skin inflammation, eventually promoting the proliferation of keratinocytes.^[3]^ Throughout the process, the interactions among dendritic cells, T cells, keratinocytes, neutrophils, and cytokines released by immune cells may promote the occurrence and persistence of the characteristic skin inflammation in psoriasis.^[4,5]^ Given the chronic and recurrent nature of psoriasis, which is difficult to cure, the primary treatment objective is to control clinical symptoms and gradually improve the quality of life for patients.^[6]^ Systemic treatments for psoriasis include phototherapy (e.g., NU-UVB, PUVA), conventional medications (e.g., methotrexate, cyclosporine, and acitretin), biologics, and small-molecule targeted drugs.^[7]^ However, the effectiveness of these treatment modalities relies on long-term administration and carries risks of recurrence and exacerbation. Hence, finding new therapeutic approaches for psoriasis has become an urgent task. Identifying risk factors for psoriasis and implementing early interventions to delay its progression are effective methods for the prevention and treatment of psoriasis.

Ferritin, a primary iron storage protein, plays a pivotal role in maintaining iron homeostasis and is involved in diverse physiological and pathological processes. In clinical practice, serum ferritin (SF) concentration has been established as a reliable biomarker of total body iron stores in individuals.^[8]^ Elevated ferritin levels serve as a critical indicator of tissue iron overload.^[9]^ Iron plays a role in various physiological processes, including oxygen transport, electron transfer, and DNA and protein synthesis.^[10]^ Iron overload, particularly in hereditary conditions such as hemochromatosis, leads to the accumulation of iron in the heart, liver, and other organs, increasing oxidative stress and thereby impairing organ function.^[11]^ Moreover, iron metabolism disorders are closely associated with age-related diseases such as cardiovascular disease, neurodegenerative diseases, and diabetes.^[12,13]^ Iron metabolism is also related to psoriasis. Studies have shown that there is iron overload in the skin tissue of psoriasis patients. In a mouse model of acute skin inflammation, imbalanced iron metabolism in keratinocytes results in excessive epidermal proliferation and neutrophil recruitment.^[14]^ This indicates a potential interaction between iron metabolism and skin homeostasis in psoriasis. Overall, the relationship between iron metabolism and psoriasis is complex and requires further study to fully understand its underlying mechanisms and clinical significance.

Therefore, this study aims to explore the relationship between SF and the severity of psoriasis. This provides additional epidemiological evidence on the relationship between iron metabolism and psoriasis, which can facilitate early detection research among psoriasis risk populations.

## 2. Methods

### 2.1. Study design

A total of 105 patients diagnosed with psoriasis, who visited Jiujiang University Affiliated Hospital between January 2018 and July 2019, were included in the study. The control group consisted of 52 healthy individuals undergoing routine health checkups during the same period. General clinical data, laboratory tests, echocardiography, and other relevant indicators were collected from participants. The study received ethical approval from the local ethics committee (No. jjuhmer-a-2019-1203) and informed consent was obtained from all participants.

#### 2.1.1. Inclusion criteria

Clinical cases diagnosed with psoriasis who were willing to participate in the study, aged between 18 and 60 years, without gender restrictions.

#### 2.1.2. Exclusion criteria

Patients who were unwilling to participate, noncompliant patients, or those below 10 years and above 80 years. Additionally, patients with acute illnesses such as fever, acute abdomen, or pregnancy were excluded. Furthermore, patients with rheumatoid arthritis accompanied by positive rheumatoid factor, malignant tumors, deep fungal or gonococcal infections, liver or kidney involvement, autoimmune diseases, cardiovascular diseases, or uncontrolled diabetes were excluded. Patients who had received systemic or oral steroid treatments within the past 6 months were also excluded from the study.

### 2.2. Data collection

The age, gender, weight, height, duration of psoriasis, and Psoriasis Area and Severity Index (PASI) were recorded for each patient. Body mass index (BMI) was calculated as weight (kg)/height^2^ (m^2^). Cases were categorized as mild (PASI ≤ 10) and severe (PASI > 10). Self-reported information on smoking and alcohol consumption, diabetes, hypertension, additional BMI definitions, and other relevant data was collected. Daily iron intake was estimated through a semi-quantitative food frequency questionnaire. Additionally, measurements of low-density lipoprotein cholesterol, glucose, C-reactive protein (CRP), hemoglobin, and albumin were conducted routinely at the laboratory of Jiujiang University Affiliated Hospital. Fasting SF levels were measured using a chemiluminescence automated analyzer (Beckman Coulter, Inc., Brea).

### 2.3. Statistical analysis

The data from this study were analyzed using SPSS 25.0 statistical software. For normally distributed continuous data, the mean ± standard deviation (mean ± s) was used, and the differences between groups were compared using an independent samples *t* test. For continuous data that did not follow a normal distribution, the median and interquartile range [M (P25, P75)] were used, and group differences were compared using the nonparametric Mann–Whitney U test. Categorical data were described in terms of frequency (%) and compared between groups using χ2 test or Fisher exact probability method. Pearson correlation analysis was used to study the correlations between SF levels and psoriasis severity scores, duration of disease, and CRP. A receiver operating characteristic curve (ROC) was plotted, and the area under the curve was calculated to evaluate the diagnostic predictive value of SF levels for psoriasis patients, along with the calculation of sensitivity, specificity, and the optimal cutoff value. All hypothesis tests were conducted with a 2-tailed approach, and *P* < .05 was considered statistically significant.

## 3. Results

### 3.1. Baseline characteristics comparison between case and control groups

A total of 105 psoriasis patients and 52 healthy controls were analyzed. The average baseline age of the participants was 42 ± 12 years, with 62 males and 95 females. Table 1 presents the demographic characteristics and comparisons of various parameters between the case and control groups. The mean SF level in the psoriasis group (251.39 ± 114.07 ng/mL) was significantly higher than that in the control group (132.32 ± 76.25 ng/mL), with a statistically significant difference (*P* < .01). The average CRP level in the psoriasis group (25.75 ± 18.29 mg/mL) was also significantly higher than that in the control group (6.24 ± 3.31 mg/mL). There were no statistically significant differences between the psoriasis group and the control group in terms of gender, age, BMI, smoking rate, drinking rate, and iron intake. Additionally, there were no differences between the 2 groups in terms of hemoglobin, fasting glucose, and low-density lipids levels as determined by laboratory tests.

**Table 1:** Comparison of parameters between psoriasis patients and controls.

	Patients (n = 105)	Controls (n = 52)	*P*-value
Age (yr)	42 ± 11	40 ± 12	.30
Male (%)	41 (39)	21 (40)	.37
BMI (kg/m^2^)	27.4 (21.6–31.8)	26.4 (20.5–29.8)	.68
Hypertension (%)	35 (33)	15 (29)	.59
Current smokers (%)	26 (25)	10 (19)	.24
Current drinkers (%)	33 (31)	14 (23)	.59
Iron intake (mg/d)	11.50 ± 2.94	12.3 ± 3.16	.12
Hemoglobin (g/dL)	12.6 (11.2–14.5)	11.4 (10.2–14.3)	.33
CRP (mg/mL)	25.75 ± 18.29	6.24 ± 3.31	**<.01**
GFR (mL/min/1.73 m^2^)	104 (96–118)	108 (104–112)	.73
Glucose (mg/dL)	105 (91–106)	102 (82–103)	.54
LDL (mg/dL)	129 (103–149)	121 (98–125)	.48
SF (ng/mL)	251.39 ± 114.07	132.32 ± 76.25	**<.01**

The bold values represent *P*-values indicating statistical significance (*P* < .05).

BMI = body mass index, CRP = C-reactive protein, GFR = glomerular filtration ratio, LDL = low-density lipids, SF = Serum Ferritin.

### 3.2. Baseline characteristics comparison among different severity levels of psoriasis

The PASI scores ranged from 1 to 36, with an average PASI of 10.61 ± 8.04 for all psoriasis patients. Among the patients, 68 (65%) had a PASI score ≤10 (Mild group), while the remaining 37 patients (35%) had a PASI score >10 (Severe group). Table 2 provides the parameter comparisons between the Mild group and the Severe group. The mean SF level in the Mild group (297.65 ± 124.54 ng/mL) was significantly lower than that in the Severe group (224.53 ± 103.37 ng/mL), with a statistically significant difference (*P* < .01). The average CRP level in the Mild group (16.42 ± 4.24 mg/mL) was also significantly lower than that in the Severe group (42.88 ± 21.62 mg/mL), with a statistically significant difference (*P* < .01). There were no statistically significant differences between the Mild group and the Severe group in terms of disease duration, gender, age, BMI, smoking rate, drinking rate, and iron intake. Additionally, there were no differences between the 2 groups in terms of hemoglobin, fasting glucose, and low-density lipids levels as determined by laboratory tests.

**Table 2:** Disease parameters according to severity of psoriasis.

	Mild (n = 68)	Severe (n = 37)	*P*-value
PSAI	6.42 ± 3.34	14.32 ± 8.47	**<.01**
Age (yr)	42 ± 12	44 ± 11	.40
Male (%)	29 (42)	12 (32)	.40
BMI (kg/m^2^)	26.8 (22.6–30.8)	29.6 (24.8–32.5)	.48
Hypertension (%)	22 (32)	13 (35)	.83
Current smokers (%)	16 (24)	10 (27)	.81
Current drinkers (%)	20 (29)	13 (35)	.66
Iron intake (mg/d)	11.4 ± 2.5	11.8 ± 3.6	.51
Hemoglobin (g/dL)	12.2 (10.7–14.2)	13.6 (11.7–13.5)	.63
CRP (mg/L)	16.42 ± 4.24	42.88 ± 21.62	**<.01**
GFR (mL/min/1.73m^2^)	110 (95–121)	103 (98–117)	.52
Glucose (mg/dL)	103 (89–104)	107 (95–108)	.38
LDL (mg/dL)	128 (104–147)	132 (101–155)	.87
SF (ng/mL)	224.53 ± 103.37	297.65 ± 124.54	**<.01**
Duration (yr)	4.62 ± 2.67	5.28 ± 2.26	.20

The bold values represent *P*-values indicating statistical significance (*P* < .05).

BMI = body mass index, CRP = C-reactive protein, GFR = glomerular filtration ratio, LDL = low-density lipids, SF = serum ferritin.

### 3.3. Pearson correlation analysis between SF and psoriasis severity score, disease duration, and CRP

In this study, the Pearson correlation coefficient was used to establish the correlation between SF levels and PASI, disease duration, and CRP. As shown in Table 3, the results showed a significant positive correlation between SF and PASI (*R* = 0.32, *P* < .01), disease duration (*R* = 0.21, *P* = .02), and CRP (*R* = 0.28, *P* < .01).

**Table 3:** Pearson correlation analysis between SF and psoriasis severity score, disease duration, and CRP.

	*r*	*P*-value
PASI	0.32	<.01
Duration	0.21	.02
CRP (mg/L)	0.28	<.01

CRP = C-reactive protein, PASSI = Psoriasis Area Severity Index, SF = serum ferritin.

### 3.4. ROC curve analysis for SF levels

**Figure 1.**
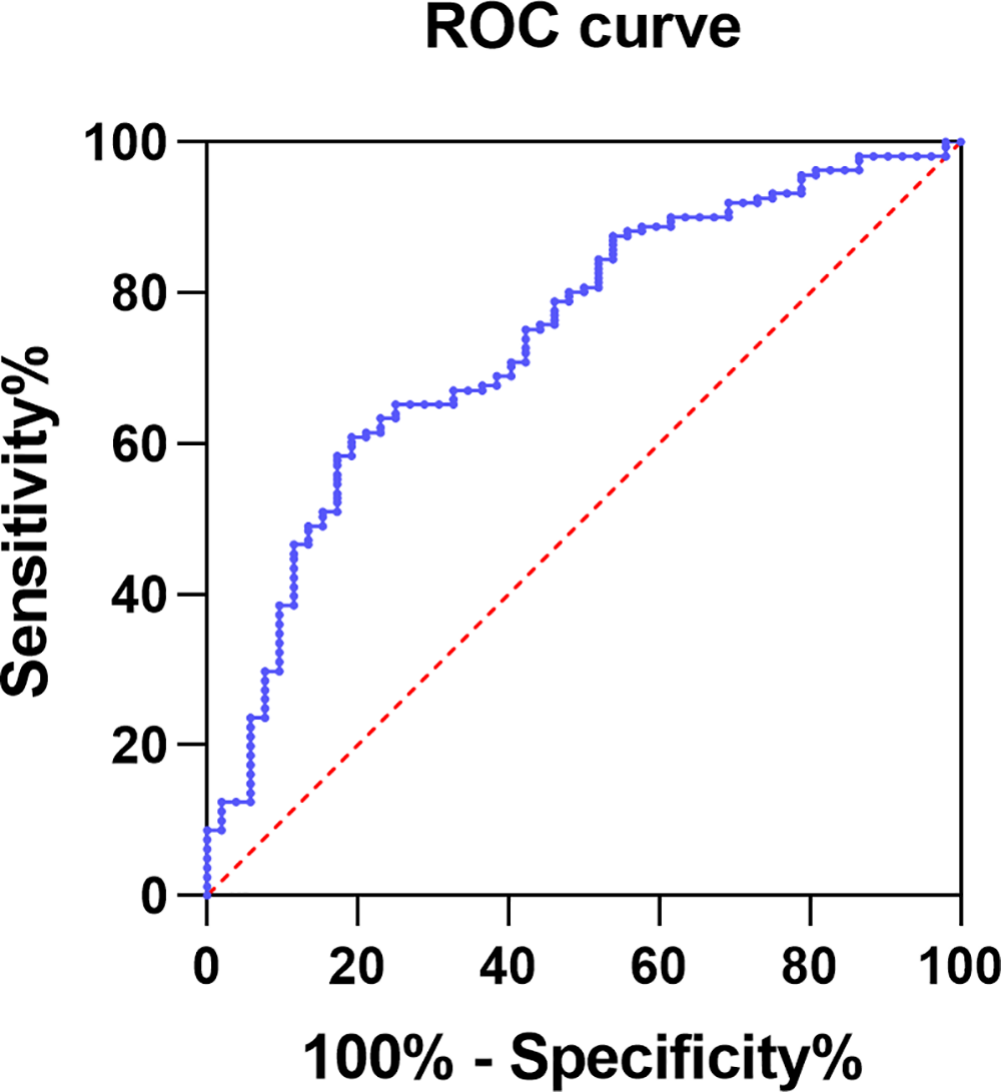
ROC curve of diagnostic value of SF levels (Control and Psoriasis).

The ROC analysis was conducted to evaluate the relationship between SF levels and the occurrence of psoriasis, with psoriasis patients assigned a value of 1 and healthy controls assigned a value of 0, as shown in Table 4 and Figure 1. The results indicated an area under the curve of 0.74 (95% CI: 0.66–0.82, *P* < .01), a sensitivity of 64%, and a specificity of 75%. The optimal cutoff value was determined to be 216.1 ng/mL. Consequently, the SF level has diagnostic value in the identification of psoriasis patients.

**Table 4:** Analysis of ROC curves for SF levels (Control and Psoriasis).

	AUC	95% CI	Cutoff value	Sensitivity(%)	Specificity (%)	*P*-value
SF	0.74	0.66–0.82	216.1	64	75	<.01

AUC = area under the curve, CI = confidence interval, ROC = receiver operating characteristic, SF = serum ferritin.

## 4. Discussion

Psoriasis is an immune-mediated chronic inflammatory skin disease that affects ~125 million people worldwide, affecting a wide range of populations and significantly impacting their quality of life.^[2]^ The pathogenesis of psoriasis is complex and remains largely unelucidated. In recent years, the critical role of iron metabolism in the pathogenesis of psoriasis has garnered increasing attention. Previous studies have shown that imbalances in iron metabolism in psoriasis mouse models lead to ferroptosis in skin cells, contributing to the progression of skin damage in psoriasis.^[15,16]^ Other studies have indicated that iron overload in the skin is involved in the progression of skin diseases such as melanoma, systemic lupus erythematosus, and vitiligo.^[17]^ Currently, there is no epidemiological evidence supporting iron overload as a risk factor for psoriasis. This cross-sectional study found a positive correlation between ferritin levels and the risk of psoriasis.

The core of the psoriasis pathogenesis is the overactivation of the adaptive immune system. In the initial stages of psoriasis, multiple cell types are activated, leading to an increase in systemic inflammatory factors, which collectively result in downstream keratinocyte proliferation, increased expression of angiogenic mediators and endothelial adhesion molecules, and the infiltration of immune cells into lesional skin.^[18]^ Clinical studies have shown that the levels of inflammatory factors in psoriasis patients are significantly higher than those in unaffected individuals.^[19,20]^ However, SF levels are significantly elevated under inflammatory and oxidative stress conditions.^[21]^ Recent study has demonstrated that elevated SF in chronic inflammation may originate from macrophage activation triggered by erythrophagocytosis.^[22]^ Ferritin levels were positively correlated with the duration of psoriasis (*R* = 0.21, *P* = .02) and also positively correlated with CRP levels (*R* = 0.28, *P* < .01) in our study suggests that prolonged cellular stress in chronic psoriasis may progressively exacerbate iron misdistribution and excessive ferritin production. However, further validation through longitudinal cohorts or interventional studies is required to elucidate the causal direction between abnormalities in iron metabolism and the progression of psoriasis. Despite adjustment for fundamental variables such as age, sex, and BMI, the weak correlation coefficients (*R* = 0.21–0.32) indicate that SF explains only a minor proportion of disease variability. Future studies should incorporate more comprehensive confounding factor models (e.g., dietary iron intake, undetected comorbidities).

Elevated ferritin levels serve as a critical indicator of tissue iron overload. Iron overload is involved in the progression of various autoimmune diseases, leading to increased systemic inflammation.^[23]^ Excessive iron accumulation regulates the functions of immune cells, causing an imbalance in immune homeostasis. Studies have found that iron overload promotes the polarization of M1 macrophages, which increases the release of inflammatory factors and exacerbates the inflammatory response.^[24,25]^ Additionally, iron overload also leads to increased mortality in hospitalized patients.^[26]^ Research has shown that ferritin levels are important risk factors for the prognosis of atherosclerosis and heart failure.^[27,28]^ One study has demonstrated that exogenous ferritin administration induces hepatic and systemic inflammation, mediated through the scavenger receptor class A member 1 (SCARA1) and subsequent neutrophil extracellular trap formation.^[29]^ This inflammatory response and cellular damage may stem from iron overload caused by excessive ferritin accumulation. Psoriasis, a severe and complex autoimmune disorder, exhibits a strong pathogenic association with both chronic inflammation and iron dysregulation.^[30]^ Recent research has identified significant epidermal iron overload in psoriasis patients, which drives neutrophil recruitment, elevated inflammatory cytokine production, and aberrant epidermal hyperproliferation.^[14]^ Our findings further demonstrate a significant positive correlation between SF levels and psoriasis severity (*R* = 0.32, *P* < .01), providing mechanistic evidence for the observed epidermal iron overload in psoriatic patients. This correlation suggests that elevated SF levels may serve as a biomarker for both disease progression and prolonged disease duration in psoriasis. Although this study measured CRP levels, the specificity of SF as an indicator of iron overload remains confounded by inflammatory status (e.g., CRP elevation may drive SF increases). Future investigations should integrate metrics such as hepcidin and transferrin saturation to differentiate between iron storage and inflammatory effects. According to our ROC analysis, SF was identified as a predictor factor for determining psoriasis, with an optimal cutoff value of 216.1 ng/mL. The SF cutoff value (216.1 ng/mL) derived from ROC analysis is cohort-specific. Verification of its clinical validity necessitates larger-scale validation in multicenter cohorts.

The main limitations of our study are as follows: SF may not fully reflect the iron overload status in PsA patients, and future research could consider other iron overload indicators to provide a more accurate assessment. The cross-sectional design limits the ability to establish a causal relationship between SF and psoriasis disease activity. Prospective studies are needed to determine whether SF is a predictor of psoriasis disease activity. Current exclusion criteria (e.g., recent steroid use) may limit the applicability of our findings to broader treated populations.

## 5. Conclusions

These cross-sectional findings demonstrate a significant positive correlation between SF levels and both psoriasis severity and disease duration, suggesting its potential utility as a biomarker for disease activity. However, translation into a clinical therapeutic target requires further support from mechanistic investigations. Large-scale prospective cohort studies are required to validate the causal relationship between systemic iron overload and psoriasis pathogenesis, particularly to disentangle inflammation-driven SF elevation from primary iron dysregulation.

## Author contributions

**Conceptualization:** Puhua Zhang, Chunxia Zhou.

**Data curation:** Yadan Cao, Fangfang Liao, Puhua Zhang.

**Formal analysis:** Linghua Liu, Fangfang Liao, Zhongcheng Hu.

**Investigation:** Puhua Zhang, Qiuhe Song, Zhongcheng Hu.

**Methodology:** Puhua Zhang, Yadan Cao, Fangfang Liao.

**Project administration:** Puhua Zhang.

**Supervision:** Chunxia Zhou, Zhongcheng Hu, Fangfang Liao.

**Writing – original draft:** Puhua Zhang, Chunxia Zhou, Qiuhe Song.

**Writing – review & editing:** Puhua Zhang, Chunxia Zhou, Qiuhe Song, Zhongcheng Hu, Linghua Liu.
